# Regulation of Proliferation and Apoptosis of Hair Follicle Stem Cells by miR-145-5p in Yangtze River Delta White Goats

**DOI:** 10.3390/genes13111973

**Published:** 2022-10-28

**Authors:** Xi Wu, Jian Wang, Yan Kang, Qiang Wang, Jingwen Qu, Xiaomei Sun, Dejun Ji, Yongjun Li

**Affiliations:** Key Laboratory for Animal Genetics & Molecular Breeding of Jiangsu Province, College of Animal Science and Technology, Yangzhou University, Yangzhou 225009, China

**Keywords:** goat, brush hair, miR-145-5p, proliferation, *DUSP6*

## Abstract

Yangtze River Delta white goats are the sole goat breed producing brush hair of high quality. The gene *DUSP6* has been extensively studied in tumor cells but rarely in hair follicle stem cells (HFSCs). Per the previous sequencing data, it was determined that *DUSP6* expression was up-regulated in superior-quality brush hair tissues, confirming it as a candidate gene associated with this trait. The targeting relationship of miR-145-5p with *DUSP6* was determined based on online database prediction and was authenticated using a dual-luciferase gene reporter assay and quantitative reverse-transcription PCR (RT-qPCR). The regulatory effect of miR-145-5p on the growth of HFSCs was determined by targeting *DUSP6* with RT-qPCR, 5-ethynyl-2′-deoxyuridine assays, Western blotting, and flow cytometry. The proliferation of HFSCs was inhibited and their apoptosis capacity was enhanced due to the presence of miR-145-5p. Therefore, it was proposed that this may have occurred through a repression effect of *DUSP6* on the MAPK signaling pathway. The regulatory network of the HFSCs can be further understood using the theoretical basis established by the findings derived from this study.

## 1. Introduction

### 1.1. Yangtze River Delta White Goat

The main goat breed belonging to the Yangtze River Delta is the Yangtze River Delta white goat, also known as Haimen goats [1]. This rare breed occurs in China and produces excellent skin, meat, and hair. These goats show early maturity, prolificacy, and roughage resistance. The goats are of medium size, with a triangular head, a small and upturned tail, slender limbs, and a dense coat of hair. The wool is white, only slightly curved, and shiny, and it is frequently used to produce writing brushes, thus termed “brush hair”. At present, brush hairs are divided into three categories, mainly according to the hair body thickness, length, and color, i.e., low-, medium-, and superior-grade hair [2]. The main difference is that the superior-quality brush hair has no medulla, while the others have a few medulla. The superior-quality brush hair, which is an extremely scarce resource, can be used to produce high-quality writing brushes.

### 1.2. Hair Follices and Hair Follice Stem Cells

In the dermal papillae of mammals, the hair root is surrounded by inner and outer hair sheaths, and the outer hair sheaths are surrounded by connective tissue cells, forming a pocket termed the hair follicle [3]. Hair follicles comprise various parts of the hair, such as the shaft, root, bulb, papilla, and hair matrix. The part of the outer sheath surrounding the root of the hair follicle that bulges out contains hair follicle stem cells (HFSCs), which are influenced by many types of hair follicle cells [4]. The regeneration of hair depends on HFSC activation [5]. HFSCs can develop into hair follicles, the epidermis, and sebaceous glands, and they can move down to the hair matrix area to mature into progenitor cells and produce hair follicles and hair shafts.

Micro-RNAs (miRNA) are small noncoding RNAs of approximately 22 nucleotides that are involved in gene regulation and thereby participate in many mechanisms presenting in the body, which include the proliferation, differentiation, and apoptosis of cells [6]. Many miRNAs have been confirmed to be related to hair follicle development, including miR-218-5p, miR-203a-3p, as well as miR-149-5p [7,8,9]. A member of the miR-145 family, miR-145-5p has been studied extensively in association with tumors, hypertension, myasthenia gravis, and psoriasis [10,11,12]. This micro-RNA can exert inhibitory effects on colorectal cancer by binding to metastasis-associated colon cancer-1 [13] and can stop the progression of breast cancer through the inhibition of SOX2 [14]. However, there is less research on the functions of miR-145-5p in hair follicles [15].

Mitogen-activated protein kinase is an important signaling pathway regarding hair growth, and dual specific phosphatase 6 (*DUSP6*) is a suppressor of extracellular signal-regulated kinase, a key gene within this pathway. *DUSP6* was considered to be a candidate gene associated with hair traits in the aforementioned goat breed that caused the generation of superior-quality brush hair, considering the pronounced differences in its expression among goats with brush hair of superior and inferior quality. The aforementioned gene was screened and identified. miR-145-5p targets the *DUSP6* gene, as determined by the bioinformatics tools for analyses and prediction functions [16]. The *DUSP6* gene was utilized to examine the potential regulatory functions of miR-145-5p in HFSCs in the specific white goat breed. Specifically, the apoptosis and proliferation status of cells, as well as cell cycle progression, were examined to elucidate the effects of miR-145-5p on these processes. The expression and inhibition of miR-145-5p were examined and its role in HFSCs was investigated for the purpose of determining its effect during the growth and development of HFSCs. The mechanism involved in the generation of brush hair of a superior quality associated with the aforementioned goat breed can be further examined using the results of this study. This research also established a basis for investigating the genetic mechanism of superior-quality brush hair.

## 2. Materials and Methods

### 2.1. Experimental Animals and Sample Collection

The Haimen National Goat Farm (Haimen, China) provided the Yangtze River Delta white goats. All experiments on the animals were carried out as per the approval and strict guidelines of the Animal Protection and Use Committee of Yangzhou University (permit number: SYXK [Su] IACUC2012-0029). Skins with brush hair of superior, as well as normal, quality were acquired from the cervical spines of 6 male goats (half-siblings, aged 6–8 months), with three samples of each skin type. The hair was shaved with scalpels, and the cervical spine skin was disinfected with 75% alcohol. The complete cervical spine skin tissues were cut to the size of 1 cm × 1 cm, cleaned with PBS, and immediately placed into liquid nitrogen, utilized for the purpose of preserving the tissue samples until their processing and analysis.

### 2.2. Total RNA Extraction and Reverse-Transcription Quantitative PCR (RT-qPCR)

The extraction of total RNA and reverse transcription into cDNA were carried out. The former process utilized the RNAiso Plus reagent (Takara, Tokyo, Japan). The TB Green^®^ Premix Ex Taq™ II (Takara) was utilized to conduct the RT-qPCR. The 2^−ΔΔCt^ methodology was employed to determine and measure the relative gene expression, with *GAPDH* as an internal control. The Primer information are in Table 1.

### 2.3. Cell Culture and Transfection for Overexpression or Knockdown

As described previously, tissues of the cervical spine of the goats were used for the isolation of HFSCs, which were then cultured. The culture fluid comprised 1 mL penicillin–streptomycin, 5 mL fetal bovine serum (Gibco, Grand Island, NY, USA), and DMEM/F12 (Gibco) to reach a final volume of 50 mL. The cells were cultured at 37 °C and 5% CO_2_ in an incubator. Afterward, the miR-145-5p mimics and inhibitors (Gene Pharma, Suzhou, China) and the corresponding control group were transfected as per the manufacturer’s instructions for the Lipofectamine 3000 kit (Invitrogen, Carlsbad, CA, USA). The cells, upon reaching 70–80% confluence, were then transfected and the Opti-MEM medium (Gibco) was utilized for the culturing and incubation of the cells for 6 h. Afterward, the culture medium was replaced with a freshly produced complete medium for cultivation for a duration of 96 h. Thereafter, stem cells were harvested at intervals of 24 h. All HFSC cultures were performed using at least three replicates. RNA oligonucleotide sequence information are in Table 2.

### 2.4. Flow Cytometry Analysis of the Cell Cycle and Apoptosis

Six-well plates (1 × 10^6^ cells/well) were utilized to culture the HFSCs in two mL of culture medium, wherein transfection took place for 48 h. Afterward, trypsin was used to digest the collected culture for 5 min and it was centrifuged at 1000× *g* for 5 min, with the supernatant being aspirated afterward. The cells were resuspended by adding approximately 1 mL of pre-cooled 1 × PBS (Solarbio, Beijing, China) and centrifuging the mixture again at 1000× *g* for 5 min. Then, 1 mL of pre-cooled ethanol (70%) was utilized to resuspend the cells and they were incubated overnight at 4 °C. PBS was utilized to wash the obtained cells, and a cell suspension using 500 μL propidium iodide (PI; Beyotime, Shanghai, China) staining buffer was formed, which was then incubated in the dark (37 °C for 30 min). The FACS LSRFortessa device (BD BioSciences, San Jose, CA, USA) was utilized in flow cytometry to probe into cell suspensions. Three independent replicate experiments were used per treatment.

Assays for apoptosis were also carried out for HFSCs by employing Annexin V-FITC/PI staining. The cells were left for 6 h for transfection and incubation and then the culture was continued with a complete medium for 48 h. Afterward, one mL 1 × PBS (pH 7.4) was utilized to wash the cells only once, and they were then digested with trypsin until they could be easily removed, placed in 1.5 mL Eppendorf tubes, and washed again with 1 mL 1 × PBS once, and 1 mL 1 × PBS was utilized to form a cell suspension. The incubation procedures of the isolated cells with Annexin V-FITC and PI, at 5 μL and 10 μL, respectively, were carried out at room temperature in a dark environment for 10 min. A flow cytometer (Beckman Coulter, Brea, CA, USA) was utilized to analyze the cells’ apoptosis.

### 2.5. 5-Ethynyl-2′-Deoxyuridine (EdU) Assay

After the cells were transfected, 24-well plates (2 × 10^5^ cells/well) were utilized to culture the cells. HFSCs were cultured for 24 h in a culture medium (0.5 mL media) that contained all the essentials and 10 µL EdU reagent. The EdU cell proliferation kit (RiboBio, Guangzhou, China) was utilized per the kit’s directions, and the staining results were obtained with inverted fluorescence microscopy. The cells were analyzed for EdU positivity using ImageJ software. Each treatment was performed using three replicates, and all imaging parameters were consistent.

### 2.6. Western Blotting (WB)

The protein from HFSCs of the different treatment groups was extracted by utilizing buffers such as 1% phenylmethylsulfonyl fluoride (Solarbio) and radioimmunoprecipitation assay protein lysis buffer to obtain the total protein. The solutions were then centrifuged at a rate of 13,000× *g* to obtain the protein fractions at 4 °C for 5 min, the protein content was quantified, and, finally, the relevant proteins were detected. The BCA protein analysis kit (Solarbio) and sodium dodecyl sulfate–polyacrylamide gel electrophoresis (SDS-PAGE) were employed to measure and filter out the different proteins, respectively. In the latter process, 20 μg protein was filtered by 8% or 10% SDS-PAGE and was then shifted to membranes of polyvinylidene fluoride. The membranes were blocked (5% skim milk, room temperature, 1 h) and overnight incubation with primary antibodies at 4 °C was carried out for the purpose of detecting relevant proteins. The primary antibodies utilized were antibodies anti-cyclin-dependent kinase 1 (*CDK1*) (MW: 34 kDa, Abcam, 1:1000 dilution), anti-proliferating cell nuclear antigen (*PCNA*) (MW: 29 kDa, Abcam, Cambridge, UK, 1:1000), anti-cyclin D2 (*CCND2*) (MW: 33 kDa, Abcam, 1:1000), anti-*BAX* (MW: 21 kDa, Abcam, 1:5000), and anti-BCL2 (*BCL2* apoptosis regulator) (MW: 26 kDa, Proteintech, Rosemont, IL, USA), and anti-*DUSP6* (MW: 42/44 kDa, Abcam, 1:1000) and anti-β-actin (MW: 42 kDa, 1:500 dilution; Abcam) were used as loading controls. After the incubation, 1 × Tris-buffered saline buffer containing Tween 20 was utilized to wash the membranes. Afterward, incubation with secondary antibodies was conducted for 1 h. These antibodies were labeled with horseradish peroxidase and included a goat-specific anti-rabbit antibody and a rabbit-specific anti-goat antibody (1:5000 dilution; Bioworld, Nanjing, China). The Super-Enhanced ECL reagent (BioSharp, Hefei, China) was employed to visualize the protein bands, whereas the Fluor Chem FC3 system (Protein-Simple, San Jose, CA, USA) was utilized to analyze the protein bands.

### 2.7. Dual Luciferase Gene Reporter Assay

The coding sequence and 3′-untranscribed region (UTR) of goat *DUSP6* downloaded from NCBI were amplified and cloned into the luciferase reporter vector psi-CHECK-2 (Promega, Madison, WI, USA). Then, the site utilized for binding by miR-145-5p was changed from GGACCAAA to GCTGGTAA to obtain the mutant *DUSP6* 3′-UTR luciferase reporter vector. The transfection of proteins, both mutant and wild-type, into 293T cells with miR-145-5p (synthesized by GenePharma, Suzhou, China) was performed to detect luciferase activity. Table 1 displays the primers utilized in this experiment.

### 2.8. Statistical Analyses

The SPSS v24 software (IBM, Armonk, NY, USA) was employed to carry out *t*-tests and one-way ANOVA. Significance is reported at *p* < 0.05.

## 3. Results

### 3.1. miR-145-5p Inhibits Goat HFSC Proliferation

**Figure 1:**
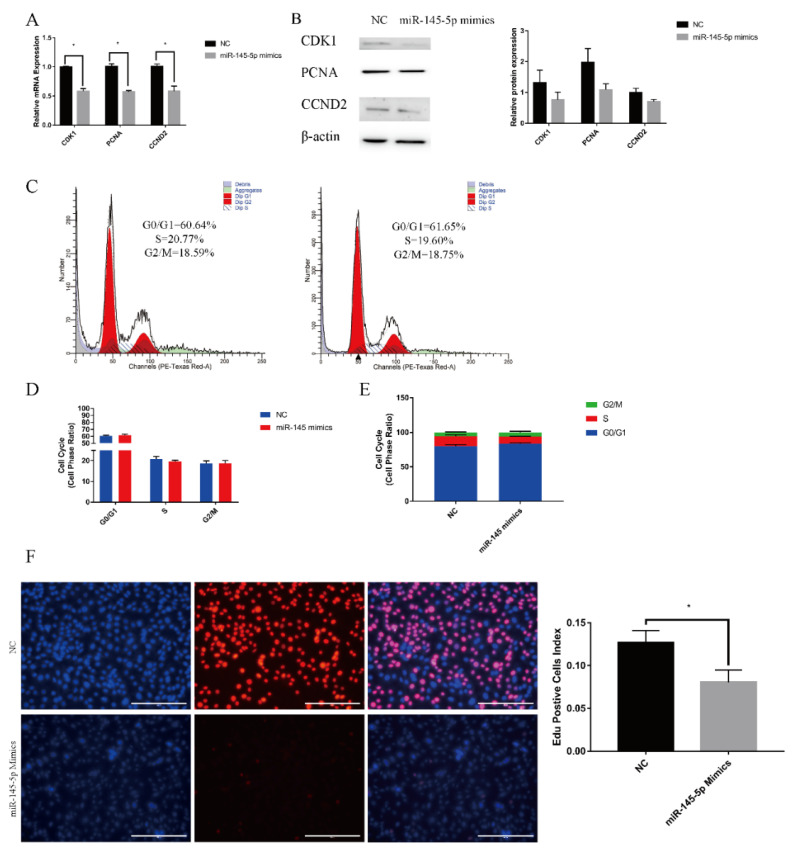
Inhibition of stem cell proliferation of goat hair follicles by miR-145-5p. (**A**) RT-qPCR quantified the expression of marker genes (*CDK1*, *PCNA*, *CCND2*) following miR-145-5p overexpression. (**B**) Western blotting showed the expression of proliferation marker genes (CDK1, PCNA, CCND2) following transfection with miR-145-5p mimics. (**C**) Cell cycle detection by flow cytometry following miR-145-5p upregulation. (**D**,**E**) The distribution of the three phases. (**F**) Distribution and rate of EdU-positive cells. No asterisk, *p* > 0.05; *, *p* < 0.05.

**Figure 2:**
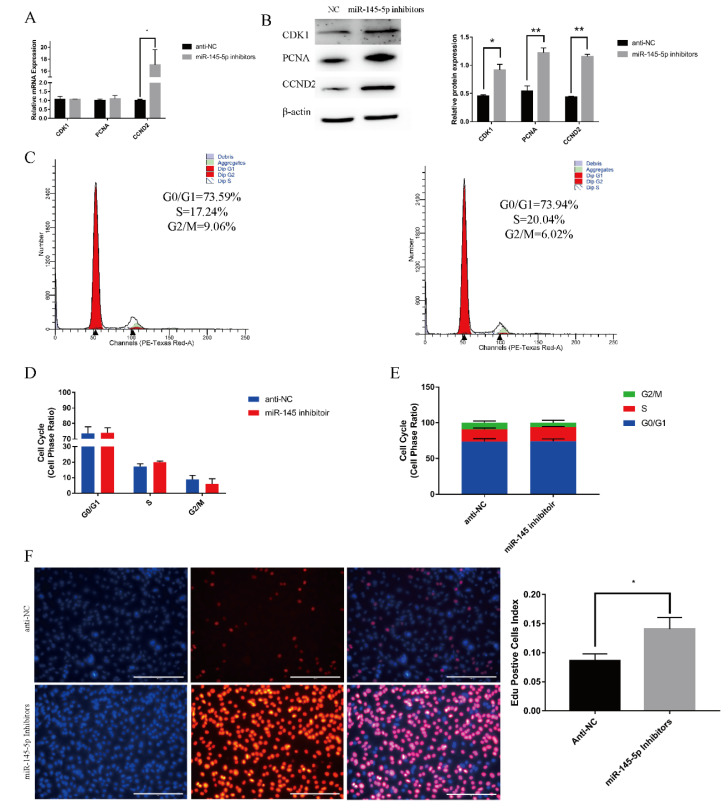
miR-145-5p knockdown promotes goat HFSC proliferation. (**A**) RT-qPCR quantified the expression of marker genes (*CDK1*, *PCNA*, *CCND2*) following miR-145-5p downregulation. (**B**) Western blotting showed the proliferation-associated marker genes’ expression levels (CDK1, PCNA, CCND2) following transfection with miR-145-5p inhibitors. (**C**) Cell cycle detection by flow cytometry following miR-145-5p knockdown. (**D**,**E**) The distribution of the three phases. (**F**) Distribution and rate of EdU-positive cells. No asterisk, *p* > 0.05; *, *p* < 0.05; **, *p* < 0.01.

To investigate the regulatory function of miR-145-5p on the proliferation of Yangtze Delta white goat HFSCs, knockdown experiments and overexpression of miR-145-5p were performed. In terms of proliferation, RT-qPCR demonstrated that the overexpression of the concerned miRNA (miR-145-5p) significantly decreased the relative expression of marker genes associated with proliferation, such as *PCNA*, *CDK1*, and *CCND2* (Figure 1A); however, the expression of the marker genes increased in the knockdown group (Figure 2A). Concerning proteins, the WB results were consistent, and the relative protein expression of PCNA, CDK1, and CCND2 was reduced in the overexpression group (Figure 1B). Similar to the relative mRNA levels, the relative protein levels were significantly increased in the knockdown group (Figure 2B). Flow cytometry showed a reduction in S-phase cells and an increasing trend in G0/G1 phase cells in the overexpression group. In the knockdown group, a decrease in S-phase cells and a decreasing trend in G0/G1-phase cells were observed (Figure 2C–E). The quantity of EdU-positive cells was significantly decreased in the concerned micro-RNA upregulated group (Figure 1F) and increased in the downregulated group (Figure 2F). These results indicated that HFSC proliferation can be inhibited by the overexpression of miR-145-5p and promoted by miR-145-5p downregulation.

### 3.2. miR-145-5p Induces Goat HFSC Apoptosis

**Figure 3:**
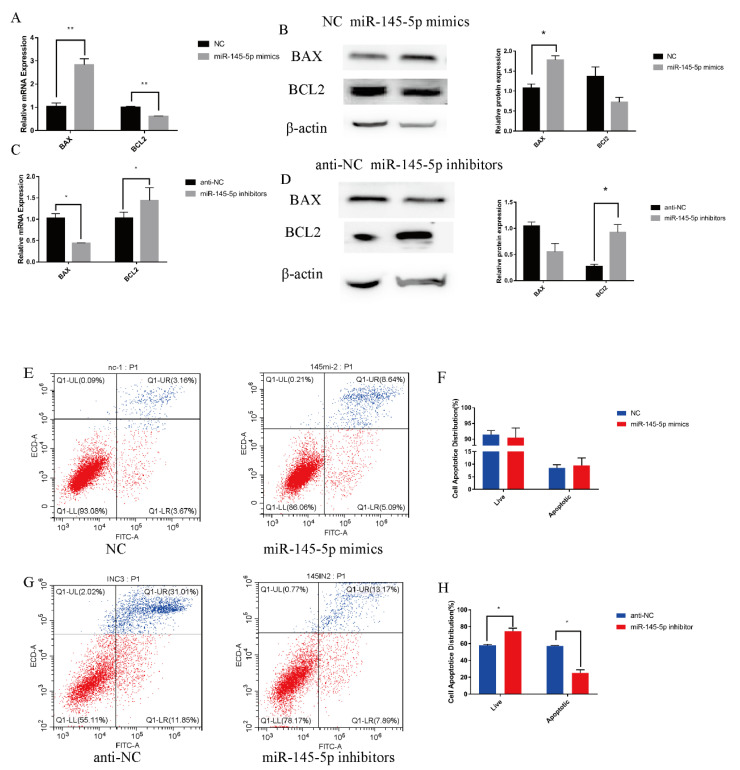
miR-145-5p regulates goat HFSC apoptosis. (**A**) RT-qPCR quantified the expression of marker genes (*BAX* and *BCL2*) following miR-145-5p upregulation. (**B**) Western blotting showed the expression of proliferation marker genes following transfection with miR-145-5p mimics. (**C**) RT-qPCR was utilized to quantify the marker genes’ expression (*BAX* and *BCL2*) following miR-145-5p downregulation. (**D**) Western blot analysis of the expression of proliferation marker genes following transfection with miR-145-5p inhibitors. (**E**–**H**) Distribution and proportion of apoptotic cells in the upgrade and knockdown groups. No asterisk, *p* > 0.05; *, *p* < 0.05; **, *p* < 0.01.

The regulatory function concerning the apoptosis of HFSCs of miR-145-5p in Yangtze Delta white goats was examined by separate knockdown and overexpression experiments. miR-145-5p overexpression considerably enhanced the relative expression of the mRNA of *BAX* and significantly decreased the expression of *BCL2*, a marker gene of apoptosis (*p* < 0.01; Figure 3A). Conversely, a considerable increase in the expression of the marker genes in the knockdown group was detected (Figure 3C). The WB results were consistent, and the relative *BCL2* protein level was reduced in the overexpression group (Figure 3B). WB showed the same result (Figure 3D). At the cellular level, the apoptotic cell rate was considerably enhanced in the mimic group and showed a decreasing trend in the inhibitor group (Figure 3E–H), which was consistent with the RT-qPCR and WB results.

### 3.3. DUSP6 Is a Direct Target Gene of miR-145-5p

**Figure 4:**
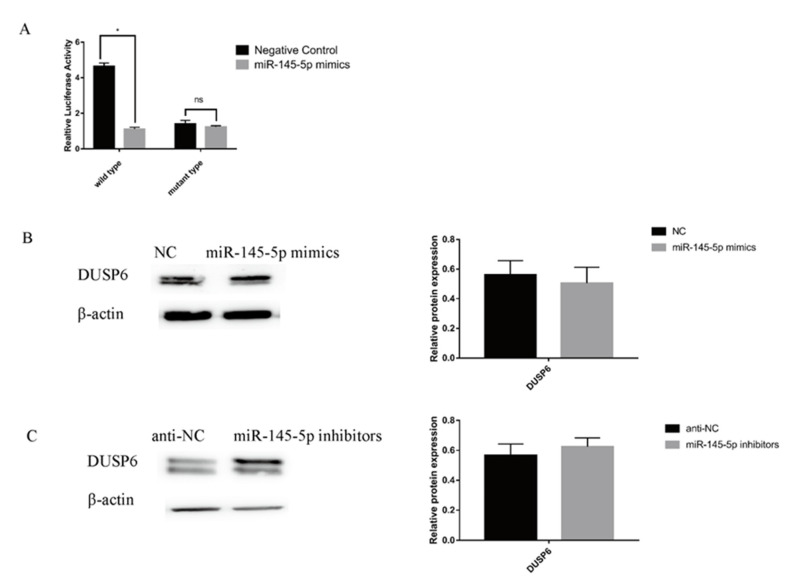
Relationship between *DUSP6* and miR-145-5p. (**A**) Dual luciferase gene reporter assay of *DUSP6* wild-type vector and *DUSP6* mutant-type vector transfected with miR-145-5p mimics. (**B**,**C**) Protein expression of *DUSP6* in the upgrade and knockdown groups. No asterisk, *p* > 0.05; *, *p* < 0.05.

The association between *DUSP6* and miR-145-5p was investigated utilizing miR-145-5p mimics transfected with *DUSP6* wild-type vector and *DUSP6* mutant-type vector transferred into 293T cells. A dual luciferase gene reporter assay regarding these miRNA mimics indicated a remarkable attenuation in luciferase activity in the wild-type vector, but no considerable variations in the activity of the mutant vector were observed (Figure 4A), indicating the direct targeting of *DUSP6* by the miRNA. At the protein level, the WB results were consistent, and the relative protein expression of *DUSP6* was reduced in the overexpression group (Figure 4B) but was increased in the knockdown group (Figure 4C). It also showed that miR-145-5p can directly target *DUSP6*.

## 4. Discussion

### 4.1. DUSP6 Plays a Key Role in MAPK Signal Pathway

The regulation of MAPK pathway activity has been associated with the protein family DUSP through the dephosphorylation of key pathway proteins [17]. The regulation of the expression of DUSP family members and their activity controls the intensity and duration of MAPK and thus defines the physiological response [18]. *DUSP6*, also known as MKP-3, belongs to the protein family DUSP and performs a key function in tumorigenesis [19]. *DUSP6* exerts crucial physiological regulatory functions in the MAPK pathway [20,21,22], and it can regulate MAPK pathway activity by binding to ERK1/2 and inactivating ERK1/2 through negative feedback regulation. *DUSP6* can also negatively regulate MAPK signaling by dephosphorylating tyrosine or serine/threonine residues on MAPK [18]. *DUSP6* regulates MAPK pathway activity and has shown a considerable influence on the development of hair follicles. Interestingly, the results confirmed *DUSP6* as a gene targeted by miR-145-5p through prediction and screening by means of bioinformatics tools, dual luciferase reporter gene analysis, RT-qPCR, and WB. The differentially expressed gene *DUSP6* was associated with the MAPK pathway of Yangtze Delta white goats concerning the quality of brush hair, linking this pathway’s role with the generation of superior-grade hair. In our previous study, *DUSP6* expression in the skin of goats of the aforementioned breed was higher in the case of brush hair of superior grade, while it was lower in goats with low-quality brush hair [23]. Furthermore, miR-145-5p can be linked to the generation of brush hair of a superior grade, as well as regulation of the development of hair follicles via the mechanism of proliferation inhibition of HFSCs in the aforementioned goat breed, as depicted by the present experimental results. The quality of the brush hair depends on the hair follicle’s development and growth. It is a very complex process involving many factors, such as the underlying biochemical and physiological processes. Therefore, as per these results, it is suggested that miR-145-5p affects the MAPK signaling pathway for hair growth and development by affecting the production of *DUSP6* in various ways.

### 4.2. miR-145-5p Targets DUSP6 to Regulate the Proliferation of HFSCs

miR-145-5p belongs to the miR-145 family, which can affect gastric cancer differentiation by directly targeting Krüppel-like factor 5 [24]. The patterns of miR-31-5p and miR-149-5P expression in HFSCs are contrary to those of miR-145-5p [1,25]; by contrast, miR-1-3p is expressed in HFSCs in a similar pattern as that of miR-145-5p. These studies suggest that different miRNAs have different regulatory effects on the growth and hair follicle development of goat HFSCs [26]. According to the results of this research, overexpression of miR-145-5p in the HFSCs of Yangtze River Delta white goats inhibited the expression of *PCNA*, *CDK1*, *CCND2*, and other proliferation marker genes and promoted the expression of *BAX* and *BCL2*. According to EdU assays, cell proliferation is inhibited after miR-145-5p overexpression. According to the cell cycle results, the number of S-phase cells in the miR-145-5p overexpression group was considerably increased over that in the control group, whereas the number of G1- and G2-phase cells showed a decreasing trend, which was, however, not significant. In comparison with the control group, the apoptosis status was increased significantly following the overexpression of miR-145-5p. This was in line with the RT-qPCR and WB results, signifying that miR-145-5p can specifically bind to *DUSP6*, resulting in reduced *DUSP6* expression. It was also consistent with the WB and double fluorescence results of the current study, suggesting a strong link between miR-145-5p and *DUSP6*. miR-145-5p thus directly targets *DUSP6*, which, as a crucial link in the MAPK signaling pathway, is important for HFSC growth and development. Furthermore, miR-145-5p overexpression lowers *DUSP6* expression levels in HFSCs, and activation of the MAPK signaling pathway is inhibited, thus affecting HFSC and hair follicle growth and development. miR-145-5p exerts a regulatory role in the Wnt/β-catenin signaling pathway, which regulates the growth and development of HFSCs [12,27,28]. It is proposed that miR-145-5p may also inhibit HFSC proliferation through the Wnt/β-catenin signaling pathway.

This study aimed to find miRNAs that regulate the formation of high-quality pencil hair traits targeting key genes (*DUSP6*) and, through research on the function and expression regulation of miRNAs, to construct the regulation mode of miR-145-5p in the formation of high-quality pencil hair traits, so as to further clarify the molecular mechanism of high-quality pencil hair traits. The results obtained can also provide a reliable basis for the accurate molecular breeding of white goats in the Yangtze River Delta. Furthermore, this study provides a relevant basis for the study of hair loss in medicine.

## 5. Conclusions

As per the results of this study, miR-145-5p can directly target *DUSP6* to further inhibit the growth and proliferation and promote the apoptosis of HFSCs in goats. The findings of this research confirmed that miR-145-5p plays a role in the regulation of HFSCs regarding apoptosis and proliferation in Yangtze River Delta white goats. The molecular processes that regulate the hair traits thus affect the quality of the brush hair and can be examined and elucidated even further utilizing the theoretical basis provided by this study.

## Figures and Tables

**Table 1:** Primer information for quantitative reverse transcription.

Gene	Primer Name	Primer Sequence (5′ to 3′)
PCNA ID:102172276	PCNA-F	ATCAGCTCAAGTGGCGTGAA
	PCNA-R	TGCCAAGGTGTCCGCATTAT
CDK1 ID:10086361	CDK1-F	AGATTTTGGCCTTGCCAGAG
	CDK1-R	AGCTGACCCCAGCAATACTT
CCND2 ID:102180657	CCND2-F	GGGCAAGTTGAAATGGAA
	CCND2-R	TCATCGACGGCGGGTAC
BCL2 ID:100861254	BCL2-F	ATGTGTGTGGAGAGCGTCAA
	BCL2-R	CCTTCAGAGACAGCCAGGAG
Bax ID:100846984	BAX-F	TTTCCGACGGCAACTTCAA
	BAX-R	TGAGCACTCCAGCCACAAA
GAPDH ID:100860872	GAPDH-F	AGGTCGGAGTGAACGGATTC
	GAPDH-R	CCAGCATCACCCCACTTGAT

**Table 2:** RNA oligonucleotide sequence information.

Genes	Sequence Name	Sequence (5′-3′)
miR-145	Negative control	UUCUCCGAACGUGUCACGUTT (sense)
		ACGUGACACGUUCGGAGAATT (antisense)
	Mimics	GUCCAGUUUUCCCAGGAAUCCCU (sense)
		GGAUUCCUGGGAAAACUGGACUU (antisense)
	Inhibitor negative control	CAGUACUUUUGUGUAGUACAA
	Inhibitors	AGGGAUUCCUGGGAAAACUGGAC

## Data Availability

The raw data supporting the conclusions of this article will be made available by the authors, without undue reservation.
